# Combining carfilzomib and panobinostat to treat relapsed/refractory multiple myeloma: results of a Multiple Myeloma Research Consortium Phase I Study

**DOI:** 10.1038/s41408-018-0154-8

**Published:** 2019-01-04

**Authors:** Jonathan L. Kaufman, Roberto Mina, Andrzej J. Jakubowiak, Todd L. Zimmerman, Jeffrey J. Wolf, Colleen Lewis, Charise Gleason, Cathy Sharp, Thomas Martin, Leonard T. Heffner, Ajay K. Nooka, R. Donald Harvey, Sagar Lonial

**Affiliations:** 1 Hematology and Medical Oncology, Winship Cancer Institute, Emory University, Atlanta, GA USA; 2 University of Chicago Medical Center, Chicago, IL USA; 3 UCSF Helen Diller Family Comprehensive Cancer Center, University of California, San Francisco, CA USA

## Abstract

Proteasome (PIs) and hystone deacetylase inhibitors (HDACis) have previously shown synergistic activity in the treatment of relapesed/refractory multiple myeloma (RRMM) patients. In this phase 1 study, we combined carfilzomib, a second generation PI, with panobinostat, a HDACi, to determine the maximum tolerated dose (MTD) of the combination (CarPan) and assess safety and efficacy among RRMM patients. Thirty-two patients (median of 4 prior lines of therapy) were enrolled. The MTD was carfilzomib 36 mg/m^2^ (on days 1, 2, 8, 9, 15, and 16) and panobinostat 20 mg (TIW, 3 weeks on/1 week off, every 28 days), administered until progression. At the MTD, the most common grade 3/4, treatment-related adverse events were thrombocytopenia (41%), fatigue (17%), and nausea/vomiting (12%). The objective response rate (ORR) and clinical benefit rate were 63% and 68%, respectively. Median progression-free survival (PFS) and overall survival (OS) for the entire population were 8 and 23 months, respectively. No differences in terms of ORR (55% vs. 57%), median PFS (months 8 vs. 7 months) and OS (24 vs. 22 months) were observed between bortezomib-sensitive and -refractory patients. CarPan proved to be a safe and effective steroid-sparing regimen in a heavily pre-treated population of MM patients. (Trial registered at ClinicalTrial.gov: NCT01549431)

## Key points

- CarPan is a highly active, steroid-sparing regimen, effective in both bortezomib-sensitive and refractory patients.
- The safety profile of CarPan, particularly thrombocytopenia and gastrointestinal adverse events, compares favorably with that of panobinostat and bortezomib.

## Introduction

Multiple myeloma (MM) is a neoplasm characterized by a clonal proliferation of plasma cells in the bone marrow and the accumulation of monoclonal protein in serum and/or urine, with related organ dysfunction. MM is the second most common hematologic malignancy, accounting for 10% of all hematologic malignancies and 1% of all cancers^1^. In the last 15 years, the introduction of the first-generation novel agents– the proteasome inhibitor (PI) bortezomib and the immunomodulatory drugs (IMiDs) thalidomide and lenalidomide– has revolutionized treatment and led to a dramatic improvement in the survival of MM patients^2,3^. However, despite the great efficacy displayed by newer agents, relapse is still inevitable and the prognosis of patients relapsing after first-generation PIs and IMiDs has been shown to be extremely poor^4^. This led to the investigation and the approval of next-generation PIs (carfilzomib, ixazomib) and the IMiD pomalidomide, as well as agents with novel mechanisms of action, such as monoclonal antibodies (elotuzumab and daratumumab) and the histone deacetylase inhibitor (HDACi) panobinostat.

Carfilzomib, a second-generation PI, is an epoxyketone that irreversibly binds to the β5 subunit of the proteasome, preventing protein degradation by the proteasome itself and thus causing an accumulation of intracellular proteins that eventually leads to cell death via apoptosis^5^. It has significant activity among patients relapsed and/or refractory (RR) to bortezomib and IMiDs, and has been approved by American and European regulatory agencies^6,7^. Currently, carfilzomib is approved with the twice-weekly schedule at a dose of 27 mg/m^2^, over a 2- to 10-min infusion, when given in combination with lenalidomide and dexamethasone (KRd), or at a dose of 56 mg/m^2^, over a 30-min infusion, when given alone or in combination with dexamethasone (Kd); nonetheless, other doses (up to 70 mg/m^2^) and schedules (once versus twice weekly) have been shown to be promising^8,9^.

Panobinostat is a pan-HDACi that exerts activity on class I, II and IV HDACs, thus regulating cell cycle, cell survival and apoptosis, and intracellular protein homeostasis^10,11^. In a phase II study, panobinostat monotherapy showed only modest activity among RRMM patients^12^. Both PIs and HDACis can regulate the metabolism on misfolded proteins, leading to their intracellular accumulation by the dual inhibition of proteasome and aggresome^13^. Based on preclinical data showing synergy between PIs and HDACis, bortezomib and panobinostat were tested in combination with dexamethasone among RRMM patients. In the placebo-controlled, phase 3 PANORAMA-1 study conducted among RRMM patients, the addition of panobinostat to bortezomib and dexamethasone (Vd) resulted in a significantly higher rate of near-complete responses (nCRs) and CRs (28% vs. 16%) as compared to placebo-Vd, and significantly prolonged median progression-free survival (PFS, 12 vs. 8 months), leading to FDA approval for RR patients who have received at least two prior therapies, including bortezomib and an IMiD^14^. Despite a remarkable clinical activity, the combination of bortezomib and panobinostat was burdened by a significant rate of grade 3/4 thrombocytopenia (64/67%) and gastrointestinal (GI) toxicity (diarrhea, 20/25%), more likely due to the overlapping toxicity profiles of the two anti-myeloma agents^14,15^.

In this light, to maximize the synergy between panobinostat and PIs, while optimizing the safety profile of the combination, panobinostat was tested, at different doses and schedules, with second-generation PIs (carfilzomib and ixazomib). In a phase 1/2 trial, carfilzomib and panobinostat (CarPan), the latter administered in a 1 week-on/1 week-off fashion, showed promising efficacy, 67% of heavily pre-treated patients achieving an objective response, and an acceptable safety profile^16^.

In order to exploit the synergy between the two drugs, we tested the administration of carfilzomib and panobinostat, both on a 3-week-on/1-week-off schedule. Herein, we report the results of a phase 1 study investigating a steroid-sparing, CarPan doublet regimen, for the treatment of RRMM patients.

## Methods

### Study population

Patients with relapsed and/or refractory MM who received at least 1 previous anti-myeloma therapy were eligible. Patients previously treated with carfilzomib were excluded from the study. RRMM was defined according to the International Myeloma Working Group (IMWG) criteria^17^. Key inclusion criteria were: age ≥ 18 years; measurable disease; Eastern Cooperative Oncology Group performance status of 0 to 2; adequate bone marrow reserves; serum creatinine clearance ≥ 30 mL per minute; adequate hepatic function (alanine aminotransferase up to 2.5 times and bilirubin up to 1.5 times of the upper normal limit). Exclusion criteria were history or presence of ventricular arrhythmias; a baseline electrocardiogram (EKG) with a QTc interval > 450 msec, uncontrolled hypertension; unstable angina or myocardial infarction within 6 months prior to enrollment, NYHA Class III or IV heart failure; impairment of GI function or GI disease that may significantly alter the absorption of panobinostat. The institutional review board at each participating center approved the study in accordance with the Declaration of Helsinki. All patients provided written informed consent. This trial was registered at Clinicatrials.gov as NCT01549431.

### Study design

This is a multi-center, phase 1 open-label study. In the dose-escalation portion of the trial, the primary endpoint was the maximum tolerated dose (MTD) of panobinostat and carfilzomib when given in combination. Patients were evaluated for dose-limiting toxicity (DLT) according to the National Cancer Institute Common Terminology Criteria for Adverse Events version 4.0. A standard 3 + 3 dose-escalation schedule was used, starting from dose level 1 with up to 4 sequential dose-escalating cohorts with 3 to 6 patients in each cohort (Table 1). A DLT was defined as any treatment-emergent toxicity attributable to at least 1 of the study drugs occurring during cycle 1. Non-hematologic DLTs included: ≥ grade 2 cardiac toxicity involving either QTc prolongation or congestive heart failure; any grade ≥ 3 toxicity; any non-hematologic toxicity requiring a dose reduction or dose interruption in cycle 1. Hematologic DLTs included: grade 4 neutropenia lasting for ≥ 7 days and febrile neutropenia; grade 4 thrombocytopenia associated with bleeding or requiring more than one platelet transfusion; and a > 7-day delay in receiving the day-1 dose of cycle 2 due to inadequate recovery of platelets ( < 75 × 10^9^/L) or other drug-related toxicity persisting from cycle 1.

**Table 1:** Dose-escalation cohorts

Cohort	Carfilzomib (mg/m^2^)^a^ on days 1, 2, 8, 9, 15, and 16 every 4 weeks	Panobinostat^b^ (mg) TIW 3 out of 4 weeks
1 (initial dose level)	27	15
2	27	20
3	36	20
4	45	20

^a^The first 2 doses of carfilzomib in cycle 1 were administered at 20 mg/m^2^ and premedication with dexamethasone 4 mg was mandatory prior to each dose of carfilzomib during cycle 1 and was thereafter administered as clinically indicated

^b^Three times a week

In the expansion phase of the trial, 12 additional patients were enrolled and treated at the MTD of panobinostat and carfilzomib to support the secondary objectives of the study including toxicity profile of the combination; objective response rate (ORR); duration of response (DOR); PFS and overall survival (OS). In the exploratory analysis, we evaluated the impact of international staging system (ISS), prior exposure to bortezomib and/or lenalidomide and disease status (refractoriness to bortezomib) on response rate, PFS and OS.

### Study procedures

Salvage treatment with CarPan consisted of 28-day cycles with twice-weekly carfilzomib (20 mg/m^2^ intravenously on day 1 and 2 cycle 1, then at a higher dose according to cohort level on days 8, 9, 15, and 16) and panobinostat (orally, three times weekly for 3 consecutive weeks 3 weeks on/1 week off. As a premedication, 4 mg of dexamethasone was mandatory prior to each dose of carfilzomib during cycle 1, and then administered as clinically indicated. Prior to each dose in cycle 1, 250 mL to 500 mL of normal saline or other appropriate IV fluid was given, as well as additional 250 to 500 mL of IV fluids as needed following carfilzomib. From cycle 2 onward, hydration before and after carfilzomib infusion was not required, unless clinically indicated. Carfilzomib was administered as an intravenous infusion over 10 min at the dose of 27 mg/m^2^ and over 30 min for doses ≥ 36 mg/m^2^. Treatment was continued until progressive disease or unacceptable toxicities.

### Statistical analysis

The sample size of the phase 1 portion was based on the scenario that cohorts (dose levels 1–4) consisted of 3 to 6 patients. The sample size of the expansion phase was based on the probability of 53.5% (confidence level) that the toxicity level of the MTD was within the range of 18 to 33%. All patients who completed at least 1 cycle of the assigned treatment or discontinued treatment during the first cycle due to toxicity were evaluable for safety; patients who started treatment and had at least one on study anti-myeloma assessment were evaluable for efficacy. Comparisons between different patient groups were investigated using Fisher’s exact test. Time to response was calculated from the start of treatment to the date of the first response (CR, very good partial response [VGPR], PR, minimal response [MR]). PFS was calculated from date of entry into the trial to the date of progression or death or the date the patient was last known to be in remission. OS was calculated from date of entry into the trial to the date of death or the date the patient was last known to be alive. Time-to-event data were analyzed using the Kaplan–Meier method. The individual effects on PFS and OS of ISS and disease refractoriness to bortezomib were evaluated using Cox proportional hazards models. Results are presented as hazard ratios (HRs) and 95% confidence intervals (95% CIs). Data were analyzed using R software (Version 3.1.1).

### Safety and response criteria

All adverse events (AEs) were assessed during each cycle and graded according to the national Cancer Terminology Criteria for Adverse Events (version 4.0)^18^. Responses were recorded at the beginning of every cycle, according to the IMWG criteria^19^; MR was defined as a decrease in the monoclonal component between 25 and 49% from baseline.

## Results

### Patient characteristics

Between February 2012 and July 2015, 32 RRMM patients were enrolled at three centers. Patient characteristics are listed in Table 2. The median age at enrollment was 66 years (range, 50–76 years). The median number of previous lines of therapy was 4 (range, 1–8), and the median time from diagnosis to enrollment was 6 years (range, 1–13 years). Twenty-seven patients (84%) were relapsed and refractory to their last line of treatment. Twenty-nine patients (91%) had previously received bortezomib; of them, 17 (53%) were bortezomib refractory. Twenty-nine (91%) patients had previously received lenalidomide; of them 10 (31%) were refractory. Eight patients (25%) were double refractory to both bortezomib and lenalidomide. Thirty out of 32 patients (94%) underwent a previous autologous stem cell transplant. At the time of data cut-off for statistical analysis, all patients had discontinued treatment, mostly due to disease progression (78%), while 19% of patients stopped treatment due to the occurrence of AEs.

**Table 2:** Patients’ characteristics

	*n* = 32 (%)
Age	
Median-years (range)	66 (50–76)
≥ 65	18 (56)
Sex	
Female	17 (53)
Male	15 (47)
Ethnicity	
Caucasian	19 (59)
African–American	12 (38)
Hispanic	1 (3)
ECOG performance status	
0	6 (19)
1	25 (78)
2	1 (3)
MM subtype	
IgG	13 (41)
IgA	8 (25)
Light chain	9 (28)
Missing	2 (6)
International staging system	
I	18 (56)
II	3 (9)
III	9 (28)
Missing	2 (6)
Bone marrow infiltration	
Plasma cells, % median (range)	30 (1–90)
Prior lines of therapy, number	
Median, range	4 (1–8)
Prior Bortezomib	29 (91)
- refractory	17 (53)
Prior Lenalidomide	29 (91)
- refractory	10 (31)
Prior pomalidomide	7 (22)
Prior anti-CD38	4 (13)
Prior autologous stem cell transplantation	30 (94)
Disease status at study entry	
Relapse and refractory	27 (84)
Relapse	5 (16)

*ECOG* Eastern Cooperative Oncology Group, *MM* multiple myeloma, *Ig* immunoglobulin, *anti-CD38* daratumumab or isatuximab

### Maximum tolerated dose

Dose levels and the observed DLTs are listed in Table 3. Four patients (1 unevaluable due to rapid disease progression) were enrolled in the first dosing cohort (carfilzomib 27 mg/m^2^, panobinostat 15 mg) with no DLT. In the second cohort, carfilzomib (27 mg/m^2^) and panobinostat (20 mg), no DLT was reported among the 3 patients evaluable (per protocol, 1 patient was not evaluable as he did not receive the treatment as planned due to missed doses not related to AEs). In cohort 3 (carfilzomib 36 mg/m^2^, panobinostat 20 mg), 1 of the 3 patients enrolled experienced a grade 3 creatinine increase and a grade 4 thrombocytopenia in cycle 1, necessitating 3 additional enrolled patients, none of whom had a DLT. Hence, the dose of carfilzomib was escalated. In cohort 4 (carfilzomib 45 mg/m^2^, panobinostat 20 mg) two DLTs were observed, a grade 4 thrombocytopenia and a grade 3 diarrhea. The MTD of the combination was then determined to be carfilzomib 36 mg/m^2^ with panobinostat 20 mg. An additional 12 patients were enrolled at the MTD, for a total of 18 patients at this dose level; of them, 16 were evaluable for efficacy.

**Table 3:** Dose-limiting toxicities

Cohort	Carfilzomib dose (mg/m^2^)	Panobinostat dose (mg)	DLT (no./patients)	DLT type
1	27	15	0/4^a^	
2	27	20	0/4^b^	
3	36	20	1/7^a^	- G4 thrombocytopenia and G3 creatinine increase
4	45	20	2/5	- G4 thrombocytopenia—G3 diarrhea

*DLT* dose-limiting toxicity, *G* grade

^a^1 Patient was not evaluable as he did not complete the first cycle due to disease progression

^b^1 Patient was not evaluable as he did not complete the assigned treatment due to lack of compliance

### Safety

Treatment-related AEs are listed in Table 4. Per protocol, 30 patients were evaluable for toxicities. Eleven patients (37%) had at least 1 treatment-related serious adverse event (SAE). Any grade hematological treatment-related AEs occurred in 29 of 30 patients (97%), while grade 3/4 hematological AEs occurred in 19 (63%) patients, including thrombocytopenia in 14 (47%), anemia in 9 (30%) and neutropenia in 6 (20%) patients. Any grade non-hematological treatment-emergent AEs were observed in 28 (93%) patients; grade 3/4 non-hematological AEs were reported in 17 (57%) patients, and the most frequent were gastrointestinal events, with nausea and vomiting, diarrhea and anorexia in 7% of patients each, fatigue (17%), and hypophosphatemia (10%). Any grade and grade 3–4 cardiac AEs occurred in 6 (20%) and 1 (3%) patients, respectively. One grade 5 AE (cardiac arrest) was observed during cycle 2 (cohort 1) in a patient with no cardiovascular comorbidities, a normal baseline EKG and no significant EKG alterations, including QT prolongation, study during treatment, and normal echocardiogram study; this event was attributed to be study related. Patients were monitored with EKG during treatment: in 4 (13%) patients a prolongation in the QT interval was registered during routine EKG, however only 1 event was reported as a grade 1 AE (3%). Any grade and grade 3/4 hypertension were observed in 7 (23%) and 2 (5%) patients.

**Table 4:** Treatment-related adverse events (≥5% of the patients)

Events, *n* (%)	All patients (*n* = 30)		MTD (*n* = 17)	
	Any grade	Grade 3–5	Any grade	Grade 3–5
Hematologic				
≥ 1 event	29 (97)	19 (63)	12 (71)	10 (59)
Anemia	12 (40)	9 (30)	5 (29)	3 (18)
Thrombocytopenia	16 (53)	6 (47)	7 (41)	7 (41)
Neutropenia	6 (20)	6 (20)	2 (12)	2 (12)
Non-hematologic				
≥ 1 event	28 (94)	17 (57)	16 (94)	10 (59)
Gastrointestinal ( ≥ 1 event)	26 (87)	7 (23)	14 (82)	5 (29)
Nausea/Vomiting	22 (73)	2 (7)	12 (71)	2 (12)
Diarrhea	17 (57)	2 (7)	8 (47)	1 (6)
Anorexia	8 (27)	2 (7)	5 (29)	1 (6)
Dysgeusia	3 (10)	–	2 (12)	–
Dyspepsia	2 (7)	1 (3)	2 (12)	1 (6)
General ( ≥ 1 event)	17 (57)	5 (17)	11 (64)	3 (18)
Fatigue	15 (50)	5 (17)	10 (59)	3 (18)
Fever	7 (23)	–	3 (18)	–
Weight loss	2 (7)	–	1 (6)	–
Neurological ( ≥ 1 event)	14 (47)	–	6 (35)	–
Insomnia	6 (20)	–	4 (24)	–
Neuropathy, sensitive	3 (10)	–	–	–
Headache	3 (10)	–	2 (12)	–
Dizziness	3 (10)	–	–	–
Cramps	2 (7)	–	1 (6)	–
Vascular ( ≥ 1 event)	7 (23)	2 (7)	4 (24)	1 (6)
Hypertension	7 (23)	2 (7)	3 (18)	1 (6)
Phlebitis	3 (10)	–	2 (12)	–
Cardiac ( ≥ 1 event)	6 (20)	2 (7)	3 (18)	1 (6)
Arrhythmias	5 (17)	–	2 (12)	–
Renal ( ≥ 1 event)	5 (17)	2 (7)	2 (12)	1 (6)
Creatinine increase	5 (17)	2 (7)	2 (12)	1 (6)
Pulmonary ( ≥ 1 event)	3 (10)	–	1 (6)	1 (6)
Dyspnea	3 (10)	–	1 (6)	1 (6)
Hepatic ( ≥ 1 event)	3 (10)	–	3 (18)	–
Bilirubin increase	2 (7)	–	2 (12)	–
Infection ( ≥ 1 event)	2 (7)	–	–	–
Dermatological ( ≥ 1 event)	2 (7)	–	2 (12)	–
Rash	2 (7)	–	2 (12)	–
Other				
Hypokalemia	4 (13)	–	4 (24)	–
Hypophosphatemia	5 (17)	2 (7)	2 (12)	1 (6)
Peripheral edema	3 (10)	–	4 (24)	–
Hypocalcemia	2 (7)	1 (3)	–	–

Percentage may not total 100% due to rounding

A low rate of any grade peripheral neuropathy was reported (10%), without any grade 3–4 events. At least 1 dose reduction of carfilzomib was necessary in 7 patients (23%) while the dose of panobinostat was reduced in 13 patients (43%). AEs leading to treatment discontinuation were thrombocytopenia (*n* = 2), acute pancreatitis (*n* = 1), cardiac arrest (*n* = 1), bowel perforation (*n* = 1), and congestive heart failure (*n* = 1).

### Efficacy

Per protocol, 30 patients were evaluable for efficacy (Table 5). Patients received a median of 8 cycles (range, 1–23 cycles) of study treatment. The ORR in the overall population was 57%; 10 patients achieved PR (33%), 5 VGPR (17%), and 2 CR (7%). Taking into account 4 patients who had a MR (13%), the clinical benefit rate (CBR) was 70%. Among patients treated at the MTD of carfilzomib and panobinostat the ORR was 63%, and 25% of patients achieved VGPR or better. The median time to at least PR was 2 months, and the median duration of response was 9 months. No differences were observed among bortezomib refractory and bortezomib-sensitive patients in terms of ORR (57% vs. 55%; *p* = 1) and at least VGPR rate (25% vs. 18%; *p* = 1). Moreover, in patients previously exposed to both bortezomib and lenalidomide, CarPan was able to induce an objective response in 48% of them, with a CBR rate of 65%.

**Table 5:** Best response with carfilzomib and panobinostat in the overall population

Best response	All	MTD	Bortezomib sensitive	Bortezomib refractory	Prior bortezomib and lenalidomide	Lenalidomide refractory	Bortezomib and lenalidomide refractory
	*n* = 30	*n* = 16	*n* = 11	*n* = 16	*n* = 23	*n* = 8	*n* = 7
	(%)	(%)	(%)	(%)	(%)	(%)	(%)
CR	2 (7)	1 (6)	1 (3)	1 (3)	2 (9)	–	–
VGPR	5 (17)	3 (19)	1 (3)	3 (10)	2 (9)	1 (13%)	–
≥ VGPR	7 (23)	4 (25)	2 (7)	4 (13)	4 (17)	1 (13%)	–
PR	10 (33)	6 (38)	4 (13)	5 (17)	7(30)	3 (38%)	3 (43%)
MR	4 (13)	1 (6)	2 (7)	2 (7)	4 (17)	1 (13%)	1 (14%)
SD	4 (13)	3 (19)	–	4 (13)	4 (7)	2 (25%)	2 (29%)
PD	5 (17)	2 (13)	3 (10)	1 (3)	4 (3)	1 (13%)	1 (14%)
ORR	17 (57)	10 (63)	6 (55)	9 (57)	11 (48)	4 (50%)	3 (43%)
CBR	21 (70)	11 (68)	8 (73)	11 (69)	15 (65)	5 (63%)	4 (57%)

Percentage may not total 100% due to rounding

*MTD* maximum tolerated dose, *CR* complete response, *VGPR* very good partial response, *PR* partial response, *MR* minimal response, *SD* stable disease, *PD* progression disease, ORR objective response rate, CBR clinical benefit rate

**Fig. 1:**
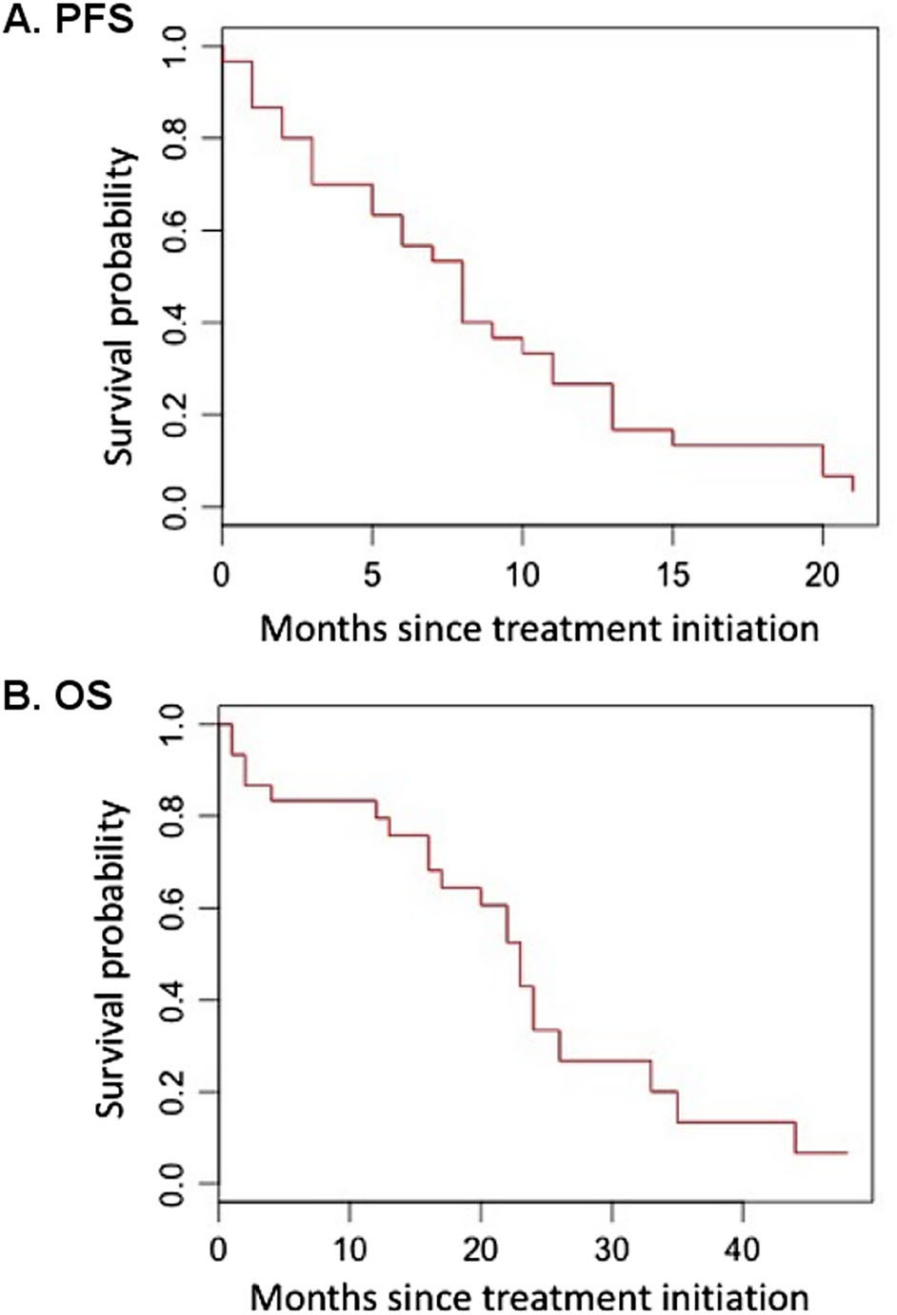
Time-to-event analysis. Kaplan–Meier progression-free survival (**a**) and overall survival (**b**) curves

No differences in ORR and at least VGPR rate were reported among patients with ISS 1 (56% and 61%, respectively) or ISS 2–3 (50% and 67%, respectively). After a median follow-up of 27 months, median PFS and OS in the overall population were 8 (95% CI: 5–11 months) and 23 months (95% CI: 16-NA months) (Fig. 1). No differences in median PFS (7 vs. 8 months; HR: 1.3, *p* = 0.5) and OS (22 vs. 24 months; HR1.8, *p* = 0.2) were noted between bortezomib refractory and sensitive patients, as well as between median PFS (8 vs. 7 months; HR: 1.9, *p* = 0.1) and OS (24 vs. 22 months; HR: 1.9, *p* = 0.15) in patients with ISS 1 as compared with ISS 2 or 3 disease.

## Discussion

In this phase 1 study, the MTD of the CarPan regimen was panobinostat 20 mg, administered 3 times weekly in a 3-week-on/1-week-off schedule, in combination with twice-weekly carfilzomib at the dose of 36 mg/m^2^. The most common AEs were hematological, mainly thrombocytopenia, and GI, including nausea, vomiting and diarrhea. In a heavily pre-treated population, in which patients have received a median of 4 prior lines of therapies and 84% of patients were relapsed and refractory to their last line of treatment, the MTD of CarPan showed promising results in terms of ORR (63%) and PFS (median, 8 months). Based on preclinical studies showing synergism between HDACis and PI (mediated by a double blockade of the intracellular protein degradation system) and evidence of a modest anti-myeloma activity displayed by single agent panobinostat, the combination of panobinostat and Vd has been tested^12,13^. The phase 2 PANORAMA-2 trial tested panobinostat, 20 mg in a 2-week-on/1-week-off schedule, with Vd in a bortezomib refractory population^15^. The addition of panobinostat to Vd was able to re-capture an objective response in 35% of the patients. Despite the evident clinical activity, panobinostat-Vd also showed a significant toxicity profile, mainly consisting of thrombocytopenia (grade 3–4, 64%), GI AEs such as diarrhea (grade 3–4, 20%), and fatigue (grade 3–4, 20%). Similar results were confirmed by the phase 3 PANORAMA-1^14^.

Carfilzomib is a second generation PI with significant activity, even among bortezomib exposed patients, that has been combined with panobinostat, at different doses and schedules, in early phase trials (NCT01301807, NCT01549431, NCT01496118)^20^. We tested escalating doses of panobinostat (up to 20 mg) administered 3 times a week in a 3-week-on/1-week-off schedule, to fit with the standard schedule of carfilzomib (twice weekly, 3 weeks on/1 week off) and maximize the synergy between proteasome and HDAC inhibition. Three out of 4 DLTs were hematological (grade 4 thrombocytopenia, *n* = 2) and GI (grade 3 diarrhea, *n* = 1) in nature. Thrombocytopenia is a well-known HDACi class-effect, due to the inhibition of maturation of megakaryocytes and the release of pro-platelets. It has been shown that it rapidly reverses withholding the drug; hence, it is possible that a different schedule might allow higher dose of panobinostat and carfilzomib^21,22^. In a phase 1/2 trial published by Berdeja et al.^16^, standard twice-weekly carfilzomib was combined with panobinostat, 3 times a week, administered every other week (1-week-on/1-week-off), in 4 week cycles. No DLTs were observed up to a dose of 30 mg for panobinostat and 45 mg/m^2^ for carfilzomib. Despite the fact that the MTD was not reached during the dose-escalation, 59% of patients in the expansion phase required dose reductions for panobinostat, and the average dose of panobinostat delivered was 23.6 mg. A second dose expansion phase was presented by Berdeja et al., with panobinostat at a dose of 20 mg (1-week-on/1-week-off) and carfilzomib at 56 mg/m^2^,^23^. Despite a toxicity profile similar to that observed in the previous cohorts, dose reductions were required for 48% of patients receiving carfilzomib and for 64% of patients receiving panobinostat; therefore, the average doses of carfilzomib and panobinostat were 48 mg/m^2^ and 14.7 mg, respectively.

The safety profile of CarPan in our trial is consistent with that reported by Berdeja et al., and better than the safety profile exhibited by panobinostat-Vd in the PANORAMA-2 trial in the rates of grade 3–4 thrombocytopenia (41% vs. 64%), diarrhea (6% vs. 20%) and fatigue (18% vs. 20%) (Table 6). The rate of grade 3–4 treatment-related hypertension in our trial was 7%, consistent with previous experience with carfilzomib when combined with dexamethasone, panobinostat, lenalidomide, and pomalidomide^8,16,20,24^. Patients were extensively monitored with serial EKGs during the trial: in 4 patients a QT prolongation was observed; however, only in 1 patient this was considered clinically significant and reported as an AE (grade 1). As expected, peripheral neuropathy occurred at a very low rate (any grade, 10%), without any serious (grade 3/4) AEs.

**Table 6:** Panobinostat-based combination in relapsed/refractory multiple myeloma patients

	Carfilzomib-panobinostat Kaufman	Carfilzomib-panobinostat Berdeja^6,23^	Panobinostat-bortezomib-dexamethasone San-Miguel^14^	Panobinostat-lenalidomide-dexamethasone Chari^25^
Panobinostat schedule	20 mg TIW, 3 weeks on/1 week off	30 mg TIW, 1 week on/1 week off	20 mg TIW, 2 weeks on/1 week off	20 mg TIW, 3 weeks on/1 week off
Prior regimens, no.	4	5	5	3
Bortezomib refractory patients	53%	36%	100%	52%
Lenalidomide refractory patients	31%	14%^a^	NA	81%
Bortezomib and lenalidomide refractory patients	25%	NA	NA	NA
ORR	63%	63%	34.5%	41%
PFS	8	8	5	7
Thrombocytopenia G3–4	41%	38%	64%	31%
Fatigue G3–4	18%	11%	20%	15%
Diarrhea G3–4	6%	11%	20%	11%
Nausea/vomiting G3–4	12%	21%	18%	0%
Discontinuation for toxicity	19%	11%	18%	NA
Dose reduction for toxicity	43%	59%	65%	41%

^a^IMiDs (thalidomide or lenalidomide) refractory

In our trial, the ORR at the MTD of CarPan (63%) compares favorably with the ORR reported with panobinostat-Vd in the PANORAMA-2 study (34.5%) and even in the PANORAMA-1 study (61%), which enrolled a less heavily pre-treated population (median number of prior lines of 1). In fact, our trial enrolled patients with a median of 4 prior therapies, half of them being bortezomib refractory^14,15^. CarPan was effective not only among bortezomib-sensitive patients (ORR: 55%; CBR: 73%), but also among bortezomib refractory patients (ORR: 57%; CBR: 69%). Of note, these results were obtained in a steroid-sparing regimen. The efficacy displayed by CarPan is consistent with that reported by Berdeja et al., in terms of ORR (63% vs. 72% in patients treated at the MTD) and median PFS (8 vs. 8 months); of note, in our trial 84% of patients were relapsed and refractory to their last regimen, a significantly higher proportion as compared to that reported by Berdeja et al. (36%)^16^.

In conclusion, we confirmed that the combination of the second-generation PI carfilzomib with panobinostat, in a two-drug, steroid-sparing regimen, is a safe and effective treatment option for RRMM patients. Future trials should compare different doses and schedules of the combination in order to optimize the treatment tolerability and enhance its efficacy.
